# Early detection of pancreatic cancer in mouse models using a novel antibody, TAB004

**DOI:** 10.1371/journal.pone.0193260

**Published:** 2018-02-20

**Authors:** Shu-ta Wu, Chandra D. Williams, Priyanka A. Grover, Laura J. Moore, Pinku Mukherjee

**Affiliations:** 1 Department of Biological Sciences, University of North Carolina at Charlotte, Charlotte, North Carolina, United States of America; 2 Department of Animal Laboratory Resources, University of North Carolina at Charlotte, Charlotte, North Carolina, United States of America; Wayne State University, UNITED STATES

## Abstract

Pancreatic ductal adenocarcinoma (PDA) is the fourth-leading cause of cancer death in the United States with a 5-year overall survival rate of 8% for all stages combined. But this decreases to 3% for the majority of patients that present with stage IV PDA at time of diagnosis. The lack of distinct early symptoms for PDA is one of the primary reasons for the late diagnosis. Common symptoms like weight loss, abdominal and back pains, and jaundice are often mistaken for symptoms of other issues and do not appear until the cancer has progressed to a late stage. Thus the development of novel imaging platforms for PDA is crucial for the early detection of the disease. MUC1 is a tumor-associated antigen (tMUC1) expressed on 80% of PDA. The goal of this study was to determine the targeting and detection capabilities of a tMUC1 specific antibody, TAB004. TAB004 antibody conjugated to a near infrared fluorescent probe was injected intraperitoneally into immune competent orthotopic and spontaneous models of PDA. Results show that fluorophore conjugated TAB004 specifically targets a) 1 week old small tumor in the pancreas in an orthotopic PDA model and b) very early pre-neoplastic lesions (PanIN lesions) that develop in the spontaneous PDA model before progression to adenocarcinoma. Thus, TAB004 is a promising antibody to deliver imaging agents directly to the pancreatic tumor microenvironment, significantly affecting early detection of PDA.

## Introduction

Incidence and mortality trends predict pancreatic cancer will become the second-leading cause of cancer related deaths by 2020 in the United States. The mean expectation of life is less than six months and there are few long-term survivors. According to the Annual Cancer Statistics Review, patients with pancreatic carcinoma have the lowest 5-year survival rate [1,2]. Poor prognosis for patients is mainly due to late diagnosis, as a result of the lack of distinct early symptoms and effective diagnostics [3]. Only 15–18% of pancreatic cancer cases are resectable, and surgery offers the only single modality for potential cure. These patients have a two-year survival rate of 20%– 40% with surgery, but despite surgical resection, local recurrence or metastasis occurs in more than 50% of the patients (predominantly liver and peritoneum). Adjuvant therapy in patients with resectable pancreatic cancer including radiation and chemotherapy is a subject of controversy with randomized trials showing contradictory results. Very often the cancer becomes resistant to such therapies. Several of these therapies also produce undesirable side effects and in some instances damage to major organs. Overall survival from PDA is only possible with surgery and adjuvant treatment when detected early [4,5]. Thus, development of an effective and targeted detection platform is essential in order to improve the survival of PDA patients. Infiltrating PDA accounts for over 95% of all exocrine pancreatic malignancies. Activating mutations in the KRAS proto-oncogene are found in over 90% of invasive PDA and are thought to represent an initiating event. Recently a transgenic mouse model has been created that expresses physiological levels of oncogenic KRAS with a glycine to aspartate substitution at codon 12 (KRAS^G12D^) in the progenitor cells of mouse pancreas. These mice develop the full spectrum of pancreatic ductal adenocarcinoma from preinvasive neoplasias (PanINs) to invasive and metastatic disease (designated as the Cre-LSL-KRAS^G12D^ or PDA mice).

Mucin-1 (MUC1), is a transmembrane protein with a heavily glycosylated extracellular domain [6]. Normal expression of MUC1 can be found on all glandular epithelial cells of the mammary gland, esophagus, stomach, duodenum, uterus, prostate, lung, and pancreas [7]. The negatively charged glycosylated extracellular domain of MUC1 in normal healthy tissues creates a physical barrier and an anti-adhesive surface, preventing pathogenic colonization [8]. However, in 80% of PDA, the extracellular domain of MUC1 is hypoglycosylated and the protein overexpressed [9]. This alteration of the structure and expression of MUC1 is associated with higher metastasis and poor prognosis [10,11] but also makes it the 2^nd^ most targetable tumor antigen [12].

We have generated the PDA.MUC1 mice by breeding the Cre-LSL-KRAS^G12D^ to a human MUC1.Tg mice (designated KCM mice) that develop the entire spectrum of PanIN lesions and adenocarcinoma mimicking the human disease [13]. We generated cell lines from these KCM mice (KCM cells) [11,14,15] and developed a novel monoclonal antibody, TAB004 (OncoTAb, Inc., Charlotte, NC), which specifically targets the hypoglycosylated/tumor-associated form of MUC1 (tMUC1) [16–19].

Using the syngeneic KCM cell lines, we demonstrate that TAB004 specifically binds to tMUC1 expressing orthotopic KCM tumors in immunocompetent mice. Further, we show that TAB004 specifically targets the pancreas in the spontaneous tumor model (the KCM mice) at the early PanIN lesion stage much before the development of invasive PDA. We show that accumulation of TAB004 is significant at the tumor site but not so at other glandular epithelial organs. TAB004 can be further developed as a diagnostics imaging tool for early detection of PDA.

## Methods

### Cell culture and generation KCM-Luc cells

KCM cell line was generated by the Mukherjee lab from spontaneous PDA tumors from KCM mice [14]. This cell line expresses both mouse Muc1 and human MUC1 and was maintained in Dulbecco’s modified Eagle’s medium (DMEM, 11965–092, Gibco, Waltham, MA). KCM-Luc cell line was generated by retroviral transduction of KCM cells with MSCV Luciferase PGK-Hygro (MSCV Luciferase PGK-hygro was a gift from Scott Lowe, Addgene plasmid # 18782, Cambridge, MA) was performed by transfecting GP2-293 cells with the MSCV Luciferase PGK-Hygro and pVSV-G vectors and using the subsequent viral supernatant to infect KCM cells. Growth media for these cell lines were supplemented with 10% fetal bovine serum (FBS, Gibco, Waltham, MA), 3.4mM ˪-glutamine, 90 units (U) per ml penicillin, and 90μg/ml streptomycin (Cellgro, Corning, Manassas, VA).

### TAB004 production and comparison

TAB004 antibody (patent US 8518405 B2 and US 9090698 B2 provided by OncoTAb. Charlotte, NC) [16,18–22]. Parental Murine TAB004 (mTAB004) is a mouse IgG1 monoclonal antibody created from a hybridoma. Chimeric TAB004 (cTAB004) is a chimeric IgG1 antibody with mouse ScFv and human Fc regions. cTAB004 was cloned into a Lake Pharma high expression stable cell line mammalian vector system (CHO cells). After two rounds of cell screening, three top performing CHO cells expressing the antibody were selected for production runs to assess the characteristics of the final stable cell line. The production of all versions of TAB-004 is currently conducted by LakePharma Inc., Belmont, CA. Comparison of mTAB004 and cTAB004 binding profiles were performed by ELISA. 100μl of 3μg/ml in 1x PBS of each antibody was used to coat 96-well ELISA plates (07-200-721, Fisher Scientific, Pittsburgh, PA) for 24 hours at 4°C. Varying concentrations of KCM cell lysate were incubated for 1 hour at room temperature. Following incubation, detection antibody conjugated to HRP was added and incubated for 1 hour at room temperature. After 100μl of TMB (PI37574, Thermo Scientific, Waltham, MA) was allowed to incubate for 30 minutes, 50μl of Stop solution (SS03, Invitrogen, Waltham, MA) was added. All steps were followed by 5x washed with 1x wash buffer.

### Conjugation of TAB004 to fluorophore

TAB004 conjugation to indocyanine green (ICG) was performed using the ICG Labeling Kit–NH_2_ (LK31-10, Dojindo Molecular Technologies, Inc., Washington, D.C.). All conjugations were performed using manufacturer protocols.

### Confocal microscopy

Tumor/pancreas sections were treated with NucBlue Fixed Cell ReadyProbes Reagent (ThermoFisher, Waltham, MA) for 5 minutes and Wheat Germ Agglutinin-488 (Molecular Probes, Waltham, MA) for 20 minutes. The slides where then washed with PBS for 5 minutes (3x) and fixed with 4% formaldehyde. Prolong Gold Antifade reagent with DAPI (P36935, Molecular Probes, Waltham, MA) was applied to mount coverslips. Images were acquired on an Olympus Fluoview FV 1000 confocal microscope.

### In vivo experiments

C57Bl/6 mice were purchased from Jackson Laboratory and housed at UNC Charlotte’s vivarium. For the orthotopic tumor model, C57/Bl6 female mice were injected in the pancreas with 5x10^5^ KCM-Luc cells and allowed to recuperate for 7 days before any experiments were performed. For tumor and antibody visualization, orthotopic KCM-Luc tumor bearing C57/Bl6 mice were injected with 125μl of Redijet D-Luciferin (760504, Perkin Elmer, Waltham, MA) intraperitoneally and imaged 25 minutes later. For groups injected with antibody, 12.5μg of IgG1 Isotype conjugated to ICG or TAB004 conjugated to ICG was injected intraperitoneally and imaged at various time points with a Perkin Elmer IVIS Spectrum. KCM mice were generated in the Mukherjee lab. This mouse model is a triple transgenic cross of LSL-KRASG12D x P48-Cre x Human MUC1.Tg that will develop PDA spontaneously and express human MUC1 in a tissue specific manner [13]. The P48-Cre mice have a tamoxifen inducible promoter and therefore, oncogenesis is initiated only when mice are treated with tamoxifen (75 mg/kg in 100ul of corn oil, 1 injection per day for 2 weeks [10 days]. T5648-1G, Millipore Sigma, St. Louis, MO) as recommended by Jackson labs (https://www.jax.org/research-and-faculty/tools/cre-repository/tamoxifen). Mice were euthanized at the end of all imaging studies. This study and all procedures were performed after approval from the Institutional Animal Care and Use Committee of UNC Charlotte.

### Image analysis

All fluorescent slide images were analyzed using Image-J (National Institutes of Health, Bethesda, Maryland). All mice and organ images were processed in Living Imagine 4.3.1 (Caliper Life Sciences, Waltham, MA).

### Immunohistochemistry

The pancreases of KCM mice were collected and samples fixed in buffered formalin and embedded in paraffin, and 5- to 6-μm–thick sections were obtained. Standard H&E staining protocol was performed and the tissue slides were then assessed by light microscopy to determine the PanIN lesions and progression to PDA at increasing disease stage. Microphotographs were taken using a DP70 camera and the Olympus Software Suite (Olympus, Waltham, MA)

## Results

### Targeting of tMUC1 in immunocompetent orthotopic PDA model with TAB004-ICG

#### Control groups

Four control groups were imaged alongside TAB004 injected groups. Radiance efficiency (RE) values were collected using the Region of Interests (ROIs) for all images.

**Fig 1:**
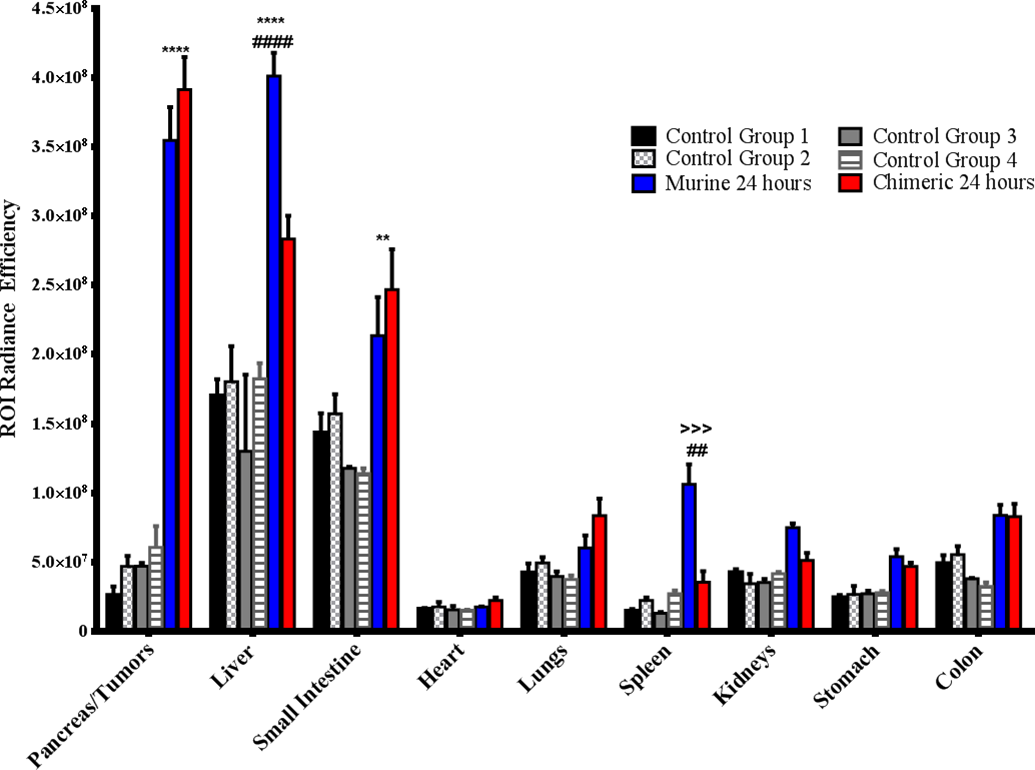
Quantification of ROI values from all imaging groups. The ROI radiance efficiency values for organs from all groups were quantified used Living Image software. * denotes significance between TAB004 (both Murine and Chimeric) and control groups, # denotes significance between Murine and Chimeric TAB004 groups, > denotes significance between Murine TAB004 and other control groups. Data shown is mean ±SEM (n = 3) and determine by 2way ANOVA with Tukey post hoc test, *p<0.05, **p<0.01, ***p<0.001, ****p<0.0001.

Control group 1 comprised of normal C57/Bl6 mice with nothing injected that had their organs imaged using the IVIS (S1 Fig). The purpose of Control group 1 was to determine background fluorescence (of ICG) levels in C57/Bl6 mice (Fig 1). Background fluorescence of the interior of the mice (in-situ) where the tumor is normally located (S1A Fig) and each organ (S1B Fig) is shown. The remaining imaging groups would use the radiance efficiency values from Control group 1 for background normalization.

Control group 2 comprised of normal C57/Bl6 with orthotopic KCM-Luc tumors (S2 Fig). This group is to determine the RE of KCM-Luc tumor by itself without injection of TAB004-ICG. The KCM cell line is a syngeneic mouse PDA cell line that expresses high levels of tMUC1 [11,19,23,24]and therefore a good target for *in vivo* TAB004 [17]. Post luciferin injection, exterior bioluminescent image in live mice confirmed the presence of KCM tumor at 7 days post KCM challenge (S2A Fig). One representative image is shown with n = 3 mice showing similar images. After imaging of the live mice, mice were euthanized and the organs of the mice in-situ photographed confirming the location of tumor in the pancreas (S2B Fig). Additionally, IVIS images confirmed that KCM tumors (S2C Fig) do not auto fluoresce. RE values of organs from Control group 2 were recorded (S2D Fig) and compared with RE values of Control group 1. There was no significant increase in RE values between in-situ and organ images of Control group 1 and 2 (Fig 1), therefore background RE values do not increase if an orthotopic tumor is present.

**Fig 2:**
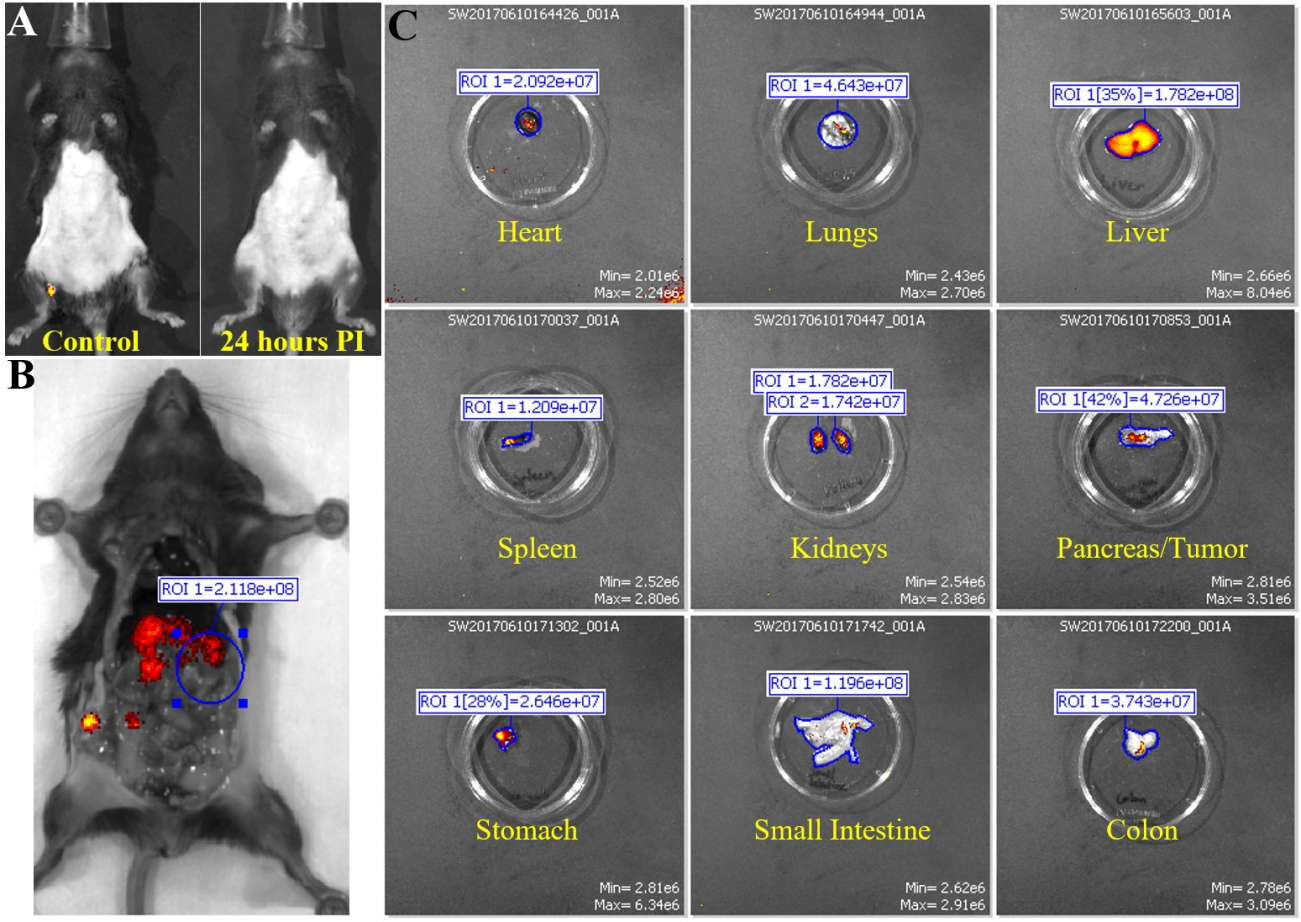
Fluorescent IVIS images of organs from mouse in control group 3. Representative images for non-tumor bearing mice injected with mouse IgG1 isotype control are shown. (A) Fluorescent IVIS image of before (control) and 24 hours post injection of IgG1 conjugated with ICG. 24 hours PI fluorescence is normalized to its own control fluorescence. (B) The mouse is imaged with filter pair ICG on the IVIS Spectrum. Background has been removed and the ROI measurements for the area where tumor would have been are shown. (C) Organs from mouse are imaged individually in the IVIS Spectrum. Intensity of the red-yellow fluorescence in ROI measurements indicate background and antibody accumulation for each organ.

**Fig 3:**
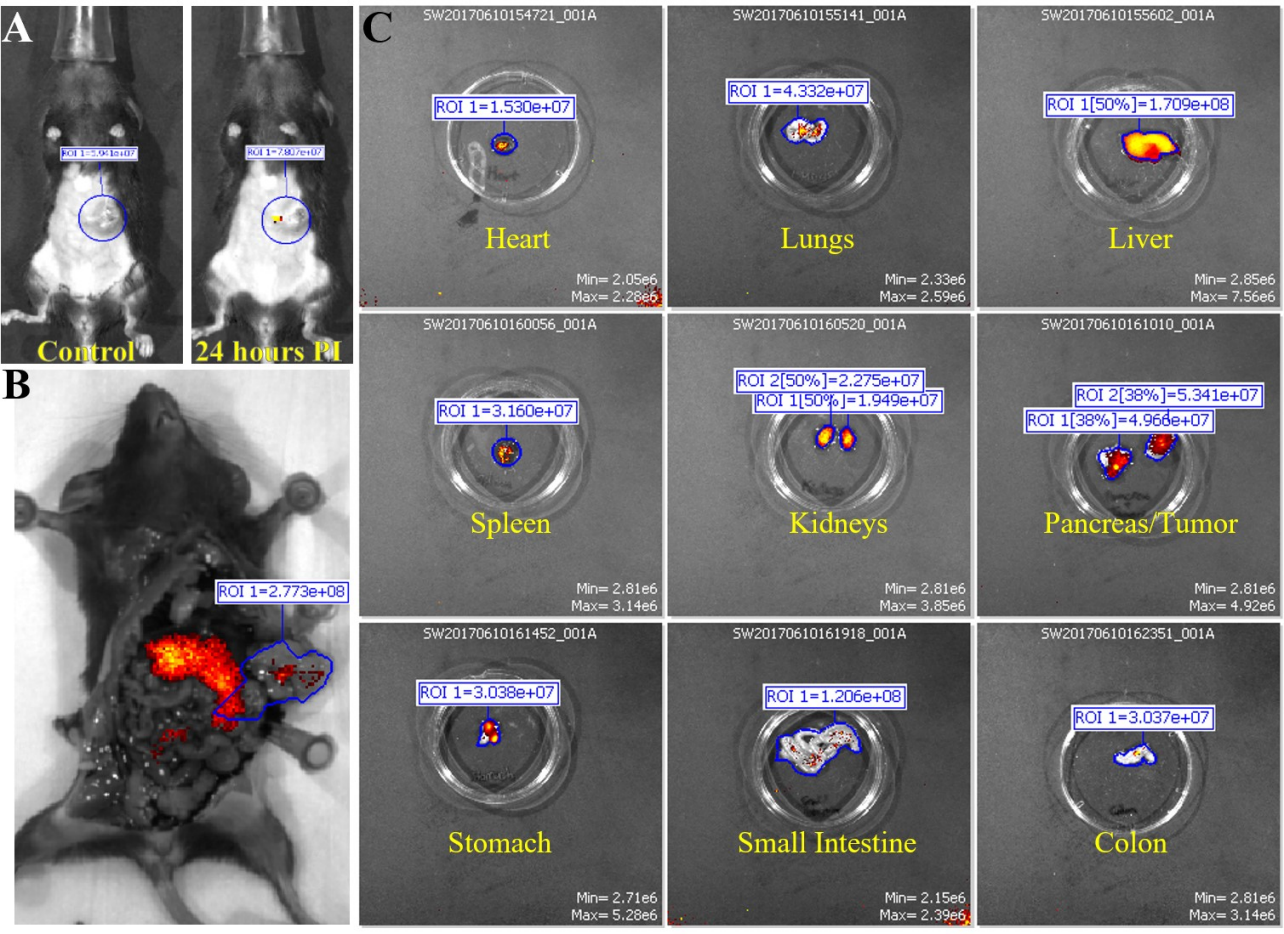
Fluorescent IVIS images of organs from mouse in control group 4. Representative images for tumor bearing mice injected wutg mouse IgG1 isotype control are shown. (A) Fluorescent IVIS image of before (control) and 24 hours post injection of IgG1 conjugated with ICG. 24 hours PI fluorescence is normalized to its own control fluorescence. (B) The mouse is imaged with filter pair ICG on the IVIS Spectrum. Background has been removed and the ROI measurements for the tumor is shown. (C) Organs from mouse are imaged individually in the IVIS Spectrum. Intensity of the red-yellow fluorescence in ROI measurements indicate background and antibody accumulation for each organ.

Control group 3 and 4. Next, we were interested in seeing if a control IgG isotype antibody conjugated to ICG would accumulate in any regions of the mice. Control group 3 consisted of normal non-tumor bearing C57/Bl6 mice that were injected with IgG1-ICG (Fig 2). Exterior ICG images taken 24 hours post injection of the IgG1-ICG show no fluorescent signal stronger than the background of the control (Fig 2A). Interior (Fig 2B) and organ (Fig 2C) ICG RE values display no significant increase over values of Control group 1 or 2 (Fig 1). Control group 4 comprised of orthotopic KCM tumor-bearing C57/Bl6 mice injected with IgG1-ICG (Fig 3). Exterior ICG images taken 24 hours after injection of the IgG1-ICG showed no significant increase in fluorescence at the tumor site (Fig 3A). Additionally, no significant increase in RE values from interior (Fig 3B) and organ (Fig 3C) images was observed between all the control groups (Fig 1). Therefore, the injection of an isotype control antibody with ICG does not increase background ICG RE values and any increase in ICG RE values seen in TAB004-ICG injected mice can be unequivocally taken as true accumulation of TAB004 at tumor region.

#### TAB004-ICG experimental groups

**Fig 4:**
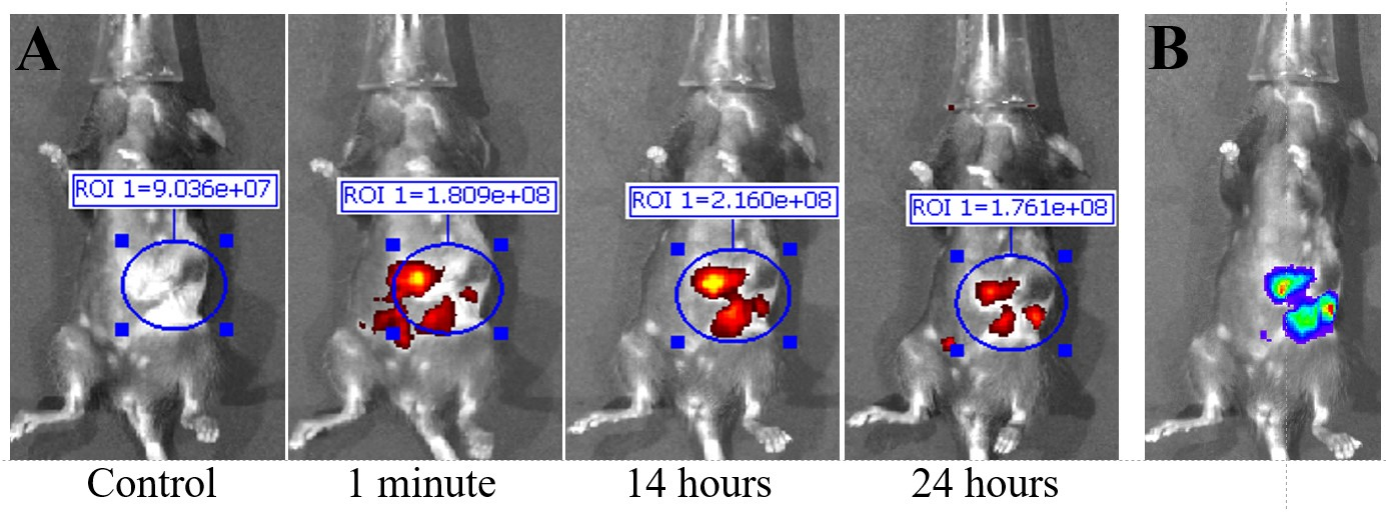
Fluorescent IVIS images taken at different time points of mouse injected with murine monoclonal TAB004. Representative images are shown. (A) Fluorescent IVIS image with filter pair ICG of before (control) 1 minutes, 14 hours, and 24 hours post injection of Parental TAB004 with ICG. Fluorescence images taken after injection are normalized to their own control fluorescence. Background has been removed and the ROI measurements for antibody fluorescence are shown. Intensity of the red-yellow fluorescence in ROI measurements indicates background and antibody accumulation. (B) Bioluminescent image of orthotopic tumor in same mouse.

**Fig 5:**
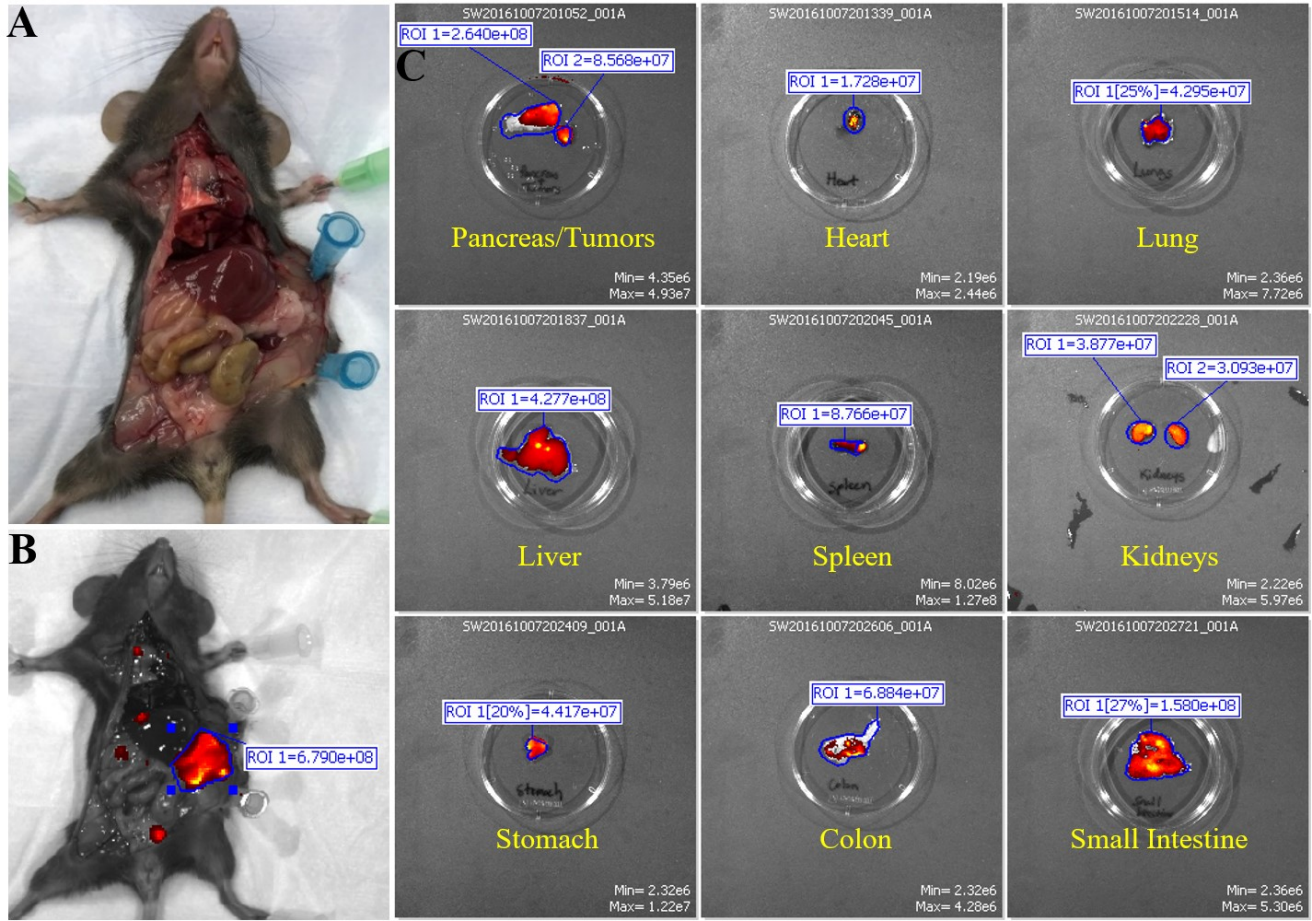
Fluorescent IVIS images of organs from mouse in injected with murine monoclonal TAB004. Representative images are shown. (A) Photograph images of mouse to show location of tumor. (B) The mouse is imaged with filter pair ICG on the IVIS Spectrum. Background has been removed and the ROI measurements for the tumor are shown. (C) Organs from mouse are imaged individually in the IVIS Spectrum. Intensity of the red-yellow fluorescence in ROI measurements indicates background and antibody accumulation for each organ.

Murine monoclonal TAB004 antibody: The first treatment group consisted of KCM tumor-bearing C57/Bl6 mice injected with mTAB004-ICG (Figs 4 and 5). Exterior ICG images taken as early as 1 minute post injection (PI) show accumulation of the mTAB004 at the tumor site (Fig 4A). The accumulation appears to increase 14 hours PI but decreases after 24 hours (Fig 4A). Exterior bioluminescent image of the same mouse confirm the location of the tumor (Fig 4B). In-situ photograph (Fig 5A) confirms the location of the tumor and IVIS images show significant accumulation of mTAB004-ICG at the tumor site (Fig 5B). The larger region of red-yellow fluorescence indicates accumulation of mTAB004 at the tumor (Fig 5B). Significant increases in RE values (Fig 5C) was observed in the pancreatic tumor, liver, small intestine, and spleen (Fig 1) which indicates the accumulation of mTAB004 selectively in those organs. RE values at the tumor site were ~ 3.5 fold higher and ~2 fold higher at the liver and small intestine site compared to the control groups (Fig 1). The increase in RE values in the liver is expected [25] from an IgG antibody. While the increase in RE values in the small intestines and spleen would suggest mTAB004 accumulation at the organs, and could be explained by some residual tumor in those organs that could not be completely dissected from the organ. Taken together, mTAB004 showed high specificity to the tumor and proves to be useful for detection of PDA by imaging.

**Fig 6:**
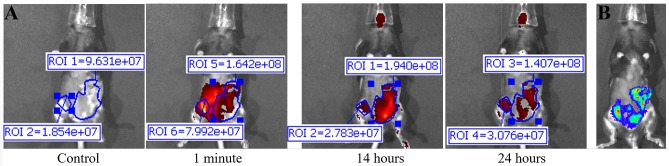
Fluorescent IVIS images taken at different time points of mouse injected with chimeric TAB004. Representative images are shown. (A) Fluorescent IVIS image with filter pair ICG of before (control) 1 minutes, 14 hours, and 24 hours post injection of Chimeric TAB004 with ICG. Fluorescence images taken after injection are normalized to their own control fluorescence. Background has been removed and the ROI measurements for antibody fluorescence are shown. Intensity of the red-yellow fluorescence in ROI measurements indicates background and antibody accumulation. (B) Bioluminescent image of orthotopic tumor in same mouse.

**Fig 7:**
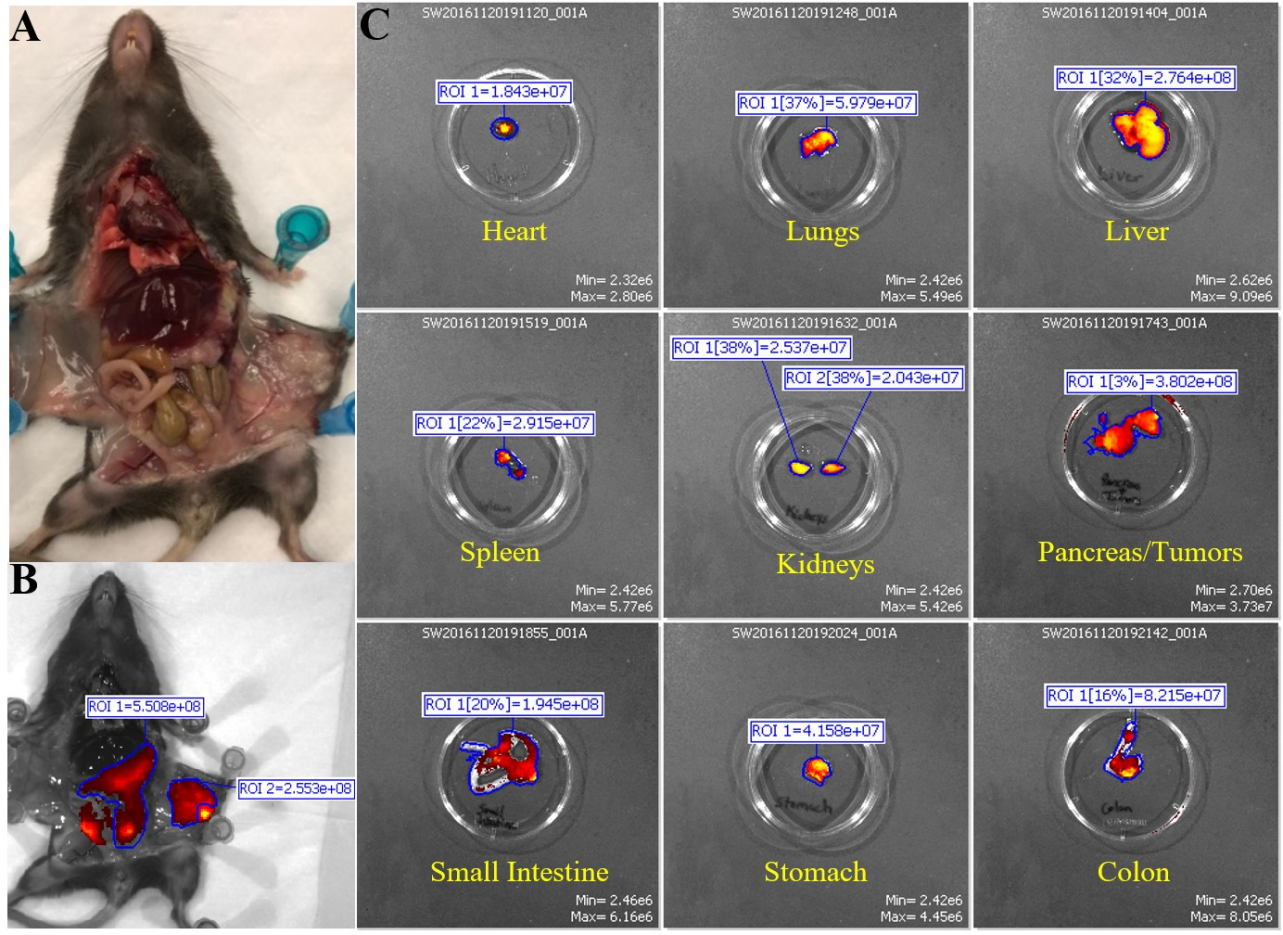
Fluorescent IVIS images of organs from mouse injected with chimeric TAB004. Representative images are shown. (A) Photograph images of mouse to show location of tumor. (B) The mouse is imaged with filter pair ICG on the IVIS Spectrum. Background has been removed and the ROI measurement for the tumor is shown. (C) Organs from mouse are imaged individually in the IVIS Spectrum. Intensity of the red-yellow fluorescence in ROI measurements indicates background and antibody accumulation for each organ.

Chimeric TAB004. Due to promising results with mTAB004, we generated a chimeric version of the antibody which shared an identical binding profile to mTAB004 in an ELISA (S3 Fig). A shift to humanize the antibody is essential for further development of any antibody-based targeted imaging for diagnostics or for targeted therapy. We acquired a chimeric version of TAB004, cTAB004, which comprises of murine antigen recognition moiety in a human IgG1 backbone. KCM orthotopic tumor-bearing C57/Bl6 mice were injected with cTAB004-ICG (Figs 6 and 7). Similar to the mTAB004-ICG (Fig 4), exterior ICG images taken as early as 1 minute post injection show accumulation of the cTAB004 at the tumor site (Fig 6A). The accumulation increases 14 hours PI but decreases post 24 hours (Fig 6A), similar to mTAB004 injected mice. Exterior bioluminescent image of the same mouse confirm the location of the tumor (Fig 6B). In-situ photograph of a cTAB004 injected mouse confirms the location of the tumor (Fig 7A). IVIS in-situ image shows accumulation in the tumor and few other organs (Fig 7B). Dissected organs were imaged separately by IVIS to calculate the RE. The larger region of red-yellow fluorescence indicates accumulation of cTAB004 at the tumor (Fig 7B). Significant increases in RE values were observed in the pancreatic tumor, liver, and small intestine (Fig 1); however, unlike mTAB004, there was minimal accumulation of the cTAB004 in the spleen. RE values at the tumor were ~ 3.5 fold higher while ~ 0.5 fold higher in the liver and ~2.5 fold higher in the small intestine compared to control RE values (Fig 1). As with mTAB004, RE values in the liver is expected to be higher [25] with an IgG antibody; however, the liver was significantly lower in the cTAB004 compared to mTAB004 injected mice, possibly due higher uptake of chimeric antibody by macrophages [26]. The RE values of cTAB004 in the small intestines is the same as the mTAB004 treatment group, where residual secondary tumor bodies could not be removed from the organ.

All experiments shown in Figs 2–7 were conducted with n = 3 mice per experimental group and representative images from 1 mouse is shown for all groups. Fig 1 shows the ROI RE values plotted for n = 3 mice per group and significance are represented as p value.

**Fig 8:**
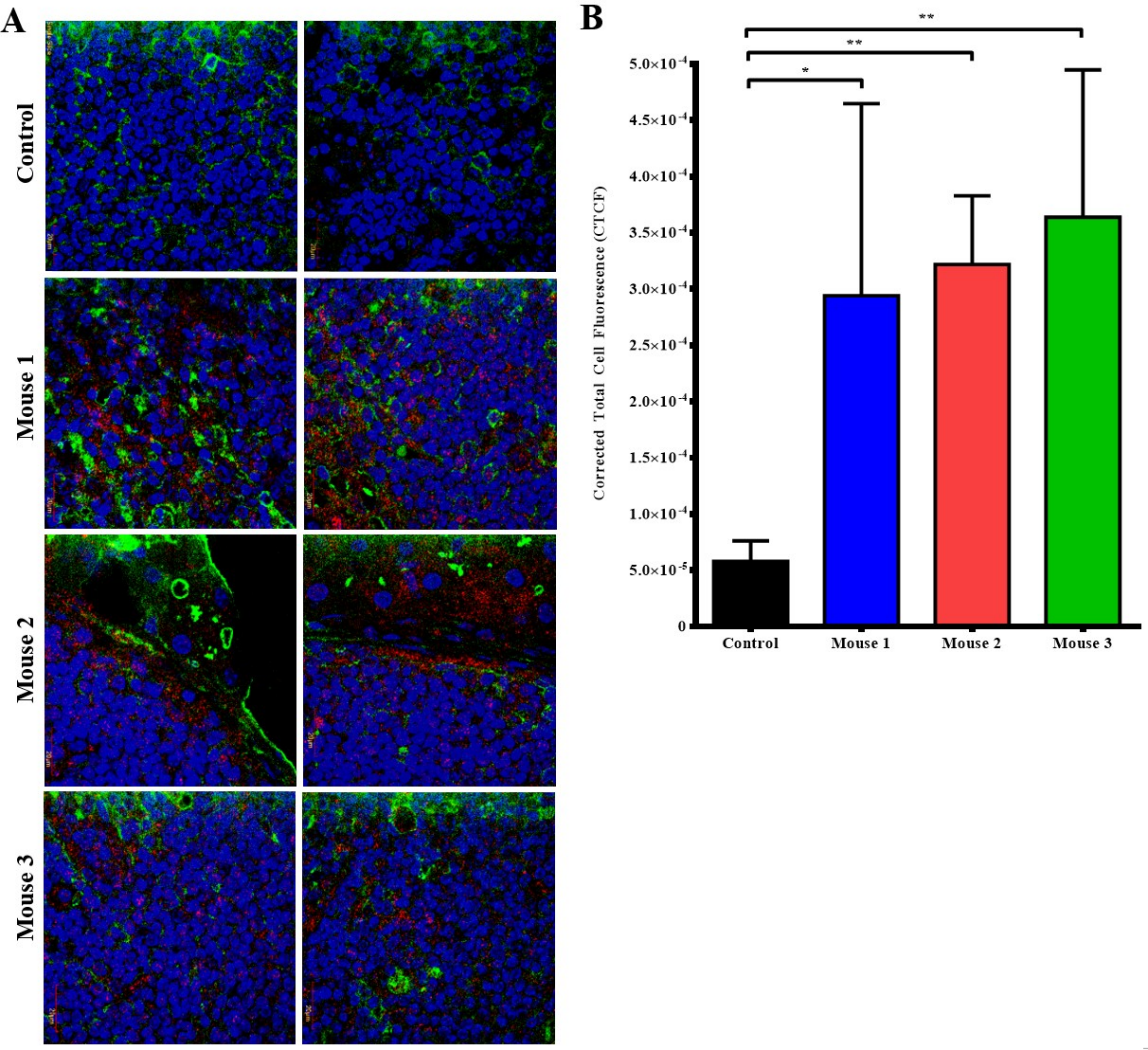
Confocal imaging of tumor sections from Chimeric TAB004 injected mice. Representative images shown. (A)Tumor sections from Chimeric TAB004 injected mice were sectioned into slides imaged. Blue = nucleus (DAPI), Green = cell membrane (Alexa Fluor 488), Red = Chimeric TAB004-ICG. (B) Quantification of fluorescent signal from Chimeric TAB004-ICG using Image J. Data shown is mean ±SEM (n = 3) and determine by unpaired t-test.*p<0.05,**p<0.01.

Tumors from 24h post cTAB004-ICG injected mice were removed, fixed, paraffin embedded, sectioned (4micron sections) and imaged under a confocal microscope to determine the cellular localization of cTAB004 ICG (Fig 8). Tumors from 3 cTAB004-ICG mice are shown alongside a control tumor from mice that were not injected with cTAB004-ICG. Tumor sections from the cTAB004-ICG injected mice display significantly more ICG fluorescent signal (red) than control tumors without TAB004 ICG (Fig 8A) in all 3 mice. The blue and green fluorescent signals are DAPI (nucleus) and wheat germ agglutinin (membrane) respectively. Red ICG fluorescence was noted within the tumor bed as well as in the edges of the tumor. Most of staining seems to be localized in the surface and cytoplasm of the tumor cells. Green fluorescence suggests the disorganized membrane staining typical of undifferentiated tumors. Quantification of the red fluorescence showed significant increase in the corrected total cell fluorescence (Fig 8B).

Overall, we have shown that cTAB004 behaves nearly identical to mTAB004 when binding to the pancreas/tumor site, with no loss in accumulation index, which was determined by the RE values (summarized in Fig 7). Expected accumulation in other organs, besides the liver, was not significantly higher than control groups. cTAB004 is highly specific for the tumor and provides rationale for further development of this platform technology for targeted imaging of PDA.

### Targeting the pancreas before PDA develops in the KCM mice

**Fig 9:**
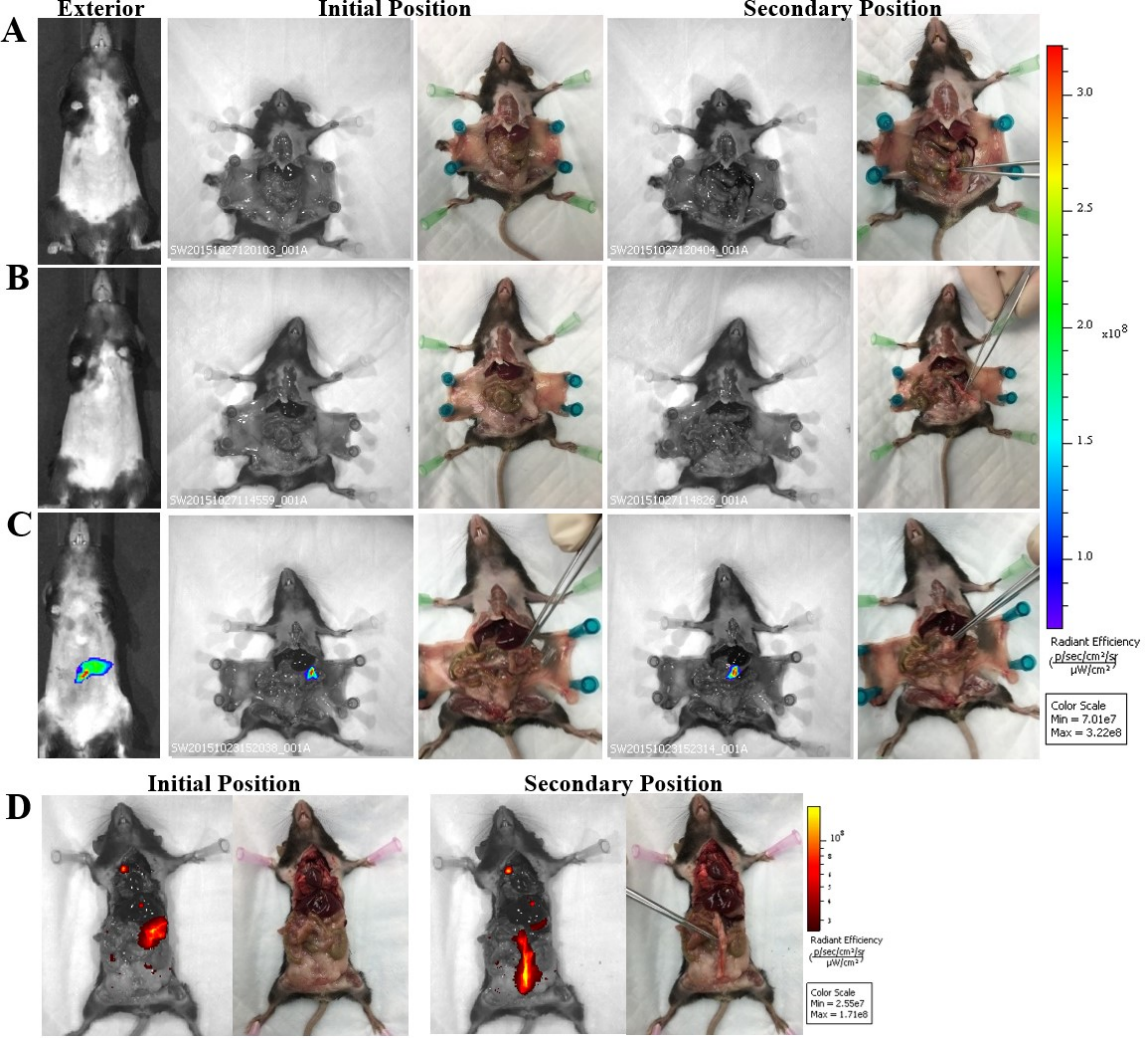
IVIS images of KC and KCM spontaneous mice (3 and 11 weeks PI of tamoxifen) injected with TAB004. Representative images are shown. Left to Right: Exterior IVIS images with ICG filter pair, IVIS image with ICG filter pair with pancreas in original position, photograph of original position, IVIS image with ICG filter pair with pancreas moved to secondary position, photo graph of secondary position. Forceps indicate location of pancreas. (A) KC mouse (lacks MUC1 transgene). (B) KCM mouse w/o tamoxifen treatment. (C) KCM 3 weeks post tamoxifen treatment. (D) KCM mouse 11 weeks post tamoxifen treatment. Rainbow fluorescence intensity indicates background and antibody accumulation in A-C. Red-yellow fluorescence intensity indicates background and antibody accumulation in D.

**Fig 10:**
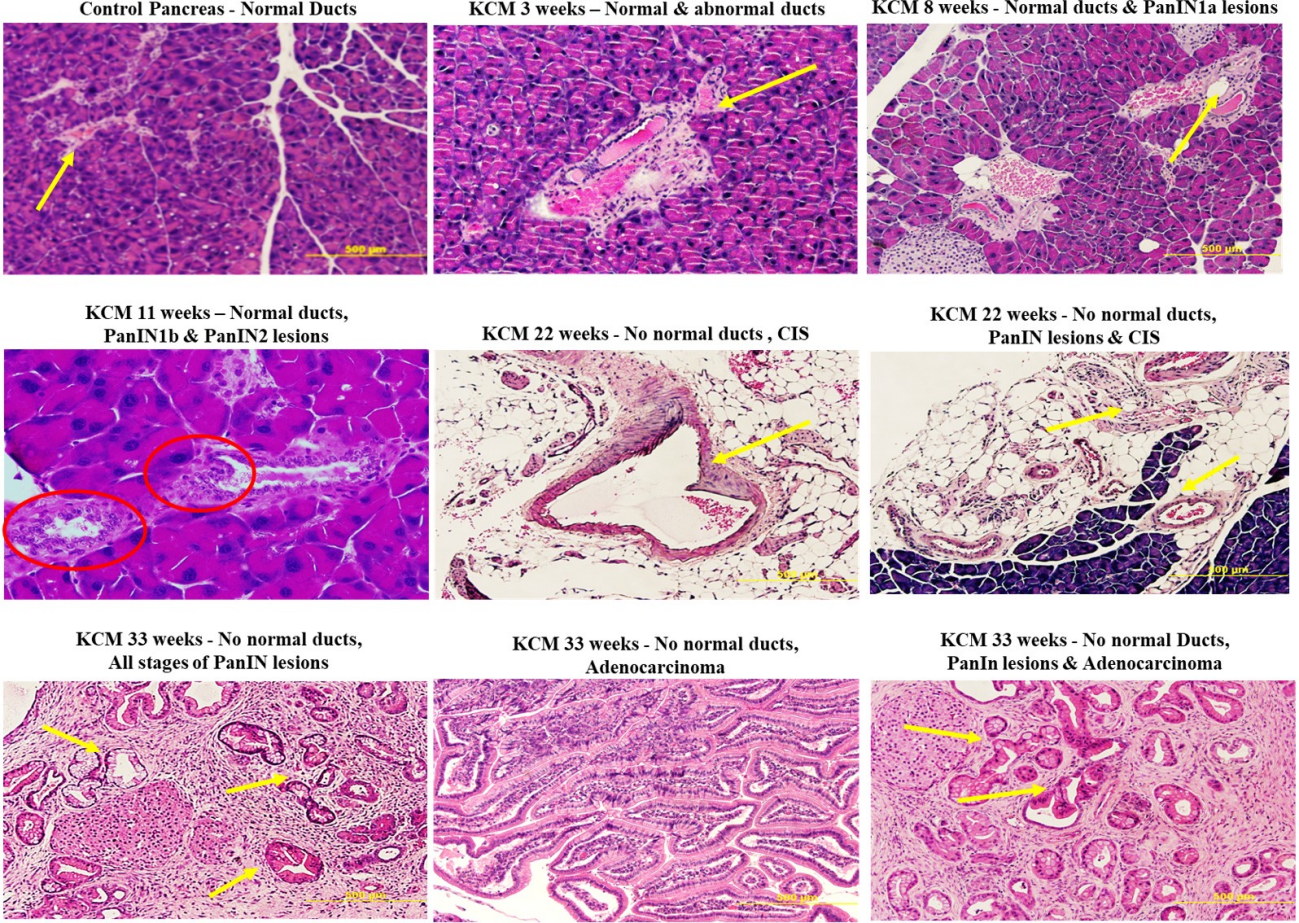
Immunohistochemistry images of pancreas from KCM mice. Representative images are shown. At different weeks post tamoxifen treatment, the progression from abnormal ducts to full adenocarcinoma is identified by H&E staining.

Although we determined the specific targeting in an orthotopic tumor model, the question remains if this antibody can be developed for early detection of pre-neoplastic lesions prior to the development of PDA. As a starting point to address this question, we generated KCM mice by crossing the LSL-KRAS^G12D^ with the tamoxifen inducible ^P48^Cre with the human MUC1.Tg mice [13]. These triple transgenic KCM mice develop spontaneous PDA when induced with tamoxifen. As controls, we generated KC mice that are double transgenic cross between LSL-KRAS^G12D^ x tamoxifen-inducible ^P48^Cre mice. KC mice do not express the human MUC1. Tamoxifen was injected in KCM and KC mice to initiate oncogenesis while control KCM mice remained without tamoxifen (therefore no initiation of oncogenesis). The 3 groups of mice (tamoxifen–induced or un-induced KCM and KC mice) were injected with cTAB004-ICG and imaged 24 hours PI (Fig 9). Both KC and KCM mice without tamoxifen induction did not display any ICG fluorescence signal in the pancreas from the exterior (Fig 9A and 9B left) or interior (Fig 9A and 9B right) IVIS images where the pancreas was imaged at its original and secondary positions. Secondary position represents moving the pancreas in situ using a forceps to confirm that any fluorescence signal is originating from the pancreas and nowhere else. As early as 3-weeks post tamoxifen induction, KCM mice showed ICG fluorescent signal over background from the exterior (Fig 9C left) and interior (Fig 9C right) IVIS images. Moving the pancreas from behind the liver and stomach displays a clearer ICG fluorescent signal over background (Fig 9C far right). Organs from these animals were also imaged with the pancreas possessing the highest level of RE over other organs, similar to the orthotopic tumor model (S3 Fig). None of the other organs showed fluorescence signal above background levels. It must be noted that in these KCM mice, all other glandular epithelial organs express normal human MUC1 but TAB004-ICG only accumulates in the pancreas post initiation of oncogenesis suggesting the high specificity of TAB004 to transformed/tumor associated form of MUC1. Based on our previous publication, we infer that at 3-weeks post completion of tamoxifen treatment, the mice have PanIN lesions 1a and b [13]. Additionally, KCM mice 11 weeks post tamoxifen induction, which develop PanIN 2 lesions at this point, were also injected with cTAB004-ICG to determine if it was possible to track disease progression (Fig 9D). By this time, the pancreas is larger and the TAB004-ICG fluorescent signal shows accumulation at the pancreas as well. Moving the pancreas from its primary location provides a clear image of the TAB004-ICG signal in the pancreas (Fig 9D). The control tamoxifen un-induced KCM and tamoxifen-induced KC mice at the same age showed no TAB004-ICG fluorescence in the pancreas or any other organ (S4 Fig). The data clearly suggests that cTAB004-ICG is binding to tMUC1at very early stages in PDA initiation (at the early PanIN stage) and is effective in tracking disease over time. All images are representative of n = 3 mice per group. Finally, H&E section of the pancreas 5, 8, 11, 22, and 33 weeks post tamoxifen injection confirms the formation of abnormal ducts at 3 weeks and PanIN lesions as early as 8 weeks (Fig 10).

## Discussion

The concept of molecularly targeted diagnostic approaches would be very valuable toward the goal of precision medicine. The ability to monitor preneoplastic lesions and progression on a molecular level not defined by the presence of a palpable tumor mass and before any overt physiologic symptoms have developed would permit early and more adequate therapeutic intervention. This study addresses this need by examining the possibility of predictive diagnostics for PDA in a genetically engineered mouse model of pancreatic cancer. tMUC1 is overexpressed in over 80% of pancreatic cancer patients [27,28] and is also expressed in pancreatic intraepithelial neoplasia (PanIN) lesions, precursors of PDA [13]. This makes it a promising target for early detection and diagnostics of PDA. Specifically, we illustrate the detection of the changes in the expression profile of the molecular biomarker tMUC1 with a noninvasive imaging approach. By focusing on early disease, i.e., the PanIN lesion, we explore a scenario in which the molecular changes in tMUC1 precede changes in anatomical and physiologic signs of tumor development. This study extends our prior work in breast cancer, which demonstrated that changes in MUC1 antigen that occur in breast cancer development could detected in vivo using TAB004 as a carrier for imaging agent [17,29]. By broadening the application of this methodology to pancreatic cancer, we move closer to establishing the value of tMUC1 as a wide-ranging cancer biomarker.

Progress has already been made in the direction of targeted cancer diagnostics. An example includes screening for the BRCA mutation for the assessment of breast cancer risk. With regard to noninvasive imaging, the development of dynamic MRI techniques, magnetic resonance spectroscopy and positron emission tomography have contributed to progress. Still, none of these technologies probes for specific molecular biomarkers expressed by cells in proportion to their potential for malignancy. Consequently, this highly specific molecular imaging approach has the potential for capturing the earliest signs of neoplastic transformation and in the future permit predictive diagnosis and response to therapy.

Our results from the orthotopic tumor model demonstrate that TAB004 is highly specific in targeting the PDA tumor and does not accumulate in other organs. In control group 1 (normal C57/Bl6 mice) and 2 (KCM-Luc tumor bearing mice–no TAB004 injected), we established background fluorescence levels for the mice and used these values to determine antibody accumulation at the tumor site and other organs. Control groups 3 (non-tumor bearing mice injected with isotype IgG1-ICG) and 4 (KCM-Luc tumor bearing mice injected with isotype IgG1-ICG) displayed no significant increase in fluorescence levels in all regions when compared to control groups 1 and 2, suggesting that from an imaging standpoint, non-targeting antibody at the chosen concentration clears from the mice in 24 hours. The significant increase in RE within the pancreas/tumor in mTAB004 and cTAB004 injected groups clearly demonstrates the retention of the targeting antibody. The livers from TAB004 injected groups displayed significant increase in RE over the control groups as well, with mTAB004 showing a greater increase over cTAB004. This appears to be a specific effect, possibly due to the presence of hepatic metastases from pancreatic cancer [30]. It also appears that TAB004 accumulates in the small intestine and spleen of mice, due to the increase in RE (Fig 7). Due to the orthotopic nature of the tumor, we have evidenced outgrowth of the primary pancreatic tumor into the surrounding organs including the spleen and small intestine due to their proximity to the pancreas. Whether this outgrowth is due to true metastasis or dissemination of tumor cells while injection is not clear at this time. Cells from the initial injection may leak out and cause many secondary tumors in the intraperitoneal space. Furthermore, we confirm that TAB004-ICG accumulates in the margins and within the pancreatic tumor bed (Fig 8). Other studies have attempted to target tMUC1 for diagnostic, imaging, and targeted therapy [17,31–33]. However, due to the non-specificity of most of the MUC1 antibodies, we believe that TAB004 can improve the specific visualization of pancreatic tumor. Furthermore, fluorescent-tagged antibody may be useful in defining the tumor margins improving patient outcome that are eligible for resection [34].

Finally, we utilized a genetically engineered mouse model that spontaneously induced human tMUC1-positive pancreatic cancer (in KCM mouse) (Figs 9 and 10). The data from the KCM mice show that TAB004 can target tMUC1 being expressed in the pancreas before PDA develops. Examination of the pancreas from KCM mice show no evidence of a primary tumor, but IHC sections from these mice show the presence of PanIN 2 lesions. PanIN 2 lesions are the first stage in the development of PDA that is associated with significant genetic and molecular changes [35]. This early detection of the PanIN lesions can be translated into early detection of PDA and significantly improve disease outcome [5,36]. Recent studies have shown there is an association of pancreatic cancer with new onset diabetes [37–39]. In some studies, diabetes was determined to be present in nearly half of the pancreatic cancer patients at diagnosis, with 75%-88% of the cases of diabetes being new onset [40,41]. It is also interesting that other studies have shown that patients with new onset diabetes have a higher chance of developing pancreatic cancer [42–44]. Perhaps the methods used in this study can be adapted for early detection of pancreatic cancer in people with new onset diabetes.

The use of ICG as an imaging agent is a limitation in this proof of concept study demonstrating the ability of TAB004 to target early stages of PDA its tumors in an immune competent model. While ICG imaging is effective in mice, it may not be as effective in humans. As an advantage, ICG has a high photon count rate, but its depth of penetration is estimated to be between 2 and 4 cm [45], limiting its utility to imaging near the surface of the patients skin or patients undergoing surgery. There is a trend to shift from fluorescent imaging to radiolabeling targeting agents such as antibodies [46] to overcome this penetrance limitation. Single-photon emission computed tomography (SPECT) and positron emission tomography (PET) are the two majors molecular imaging modalities based on the detection of radioactive decay. Both PET and SPECT do not have limits in regards to their penetration depth and the image data is highly quantifiable [47–49]. For future studies we propose to use a fully humanized version of TAB004 with radioisotope labeling to target human xenograft tumors in immune compromised mice. Presently, this is outside the scope of this study.

As tMUC1 expression has global relevance in adenocarcinomas including pancreatic cancer, this study focused on applying our imaging approach to pancreatic cancer, as it has the lowest survival rate among all common cancers.

## Supporting information

S1 Fig Fluorescent IVIS images of organs from normal C57/Bl6. Representative images are shown. (A) The mouse is imaged with filter pair ICG on the IVIS Spectrum. Background has been removed and the ROI measurement for the area where tumor would have been present is shown. (B) Organs from mouse are imaged individually in the IVIS Spectrum. Intensity of the red-yellow fluorescence in ROI measurements indicate background levels for each organ.
(TIF)

S2 Fig Fluorescent and bioluminescent IVIS images of organs from normal C57/Bl6 with orthotopic KCM-Luc tumors without TAB004 ICG injection. Representative images are shown. (A) Bioluminescent image of tumor. Rainbow indicates tumor site. (B) Photograph images of mouse to show location of tumor. (C) The mouse is imaged with filter pair ICG on the IVIS Spectrum. Background has been removed and the ROI measurements for the area where tumor is present and would have been present are shown. (D) Organs from mouse are imaged individually in the IVIS Spectrum. Intensity of the red-yellow fluorescence in ROI measurements indicates background levels for each organ.
(TIF)

S3 Fig Binding profiles of murine and chimeric TAB004. The binding profiles of mTAB004 (red) and cTAB004 (blue) were determined by ELISA and the OD values graphed against concentrations of KCM lysate.
(TIF)

S4 Fig Fluorescent IVIS images of organs from KCM mice. Representative images are shown. (A) IVIS images with ICG filter pair of organs from KCM Spontaneous mouse 3 weeks post tamoxifen induction, KCM Spontaneous mouse w/o tamoxifen, and a KC mouse. B) Organs from a KCM Spontaneous mouse 11 weeks post tamoxifen induction. Left–photograph of organs, Middle–Legend, Right–IVIS images with ICG filter pair. Intensity of the red-yellow fluorescence in ROI measurements indicates background and antibody accumulation for each organ.
(TIF)

S5 Fig Quantification of ROI values from control imaging groups. The ROI radiance efficiency values for organs from control groups were quantified used Living Image software. Data shown is mean ±SEM (n = 3), except for Mouse IgG1 in KCM group (n = 1, only 1 mouse was available for this experiment).
(TIF)
