# Supplementary figures and images for: Early detection of pancreatic cancer in mouse models using a novel antibody, TAB004
Source: PLoS One. 2018 Feb 20;13(2):e0193260. doi: 10.1371/journal.pone.0193260 (PMC5819830; doi:10.1371/journal.pone.0193260)

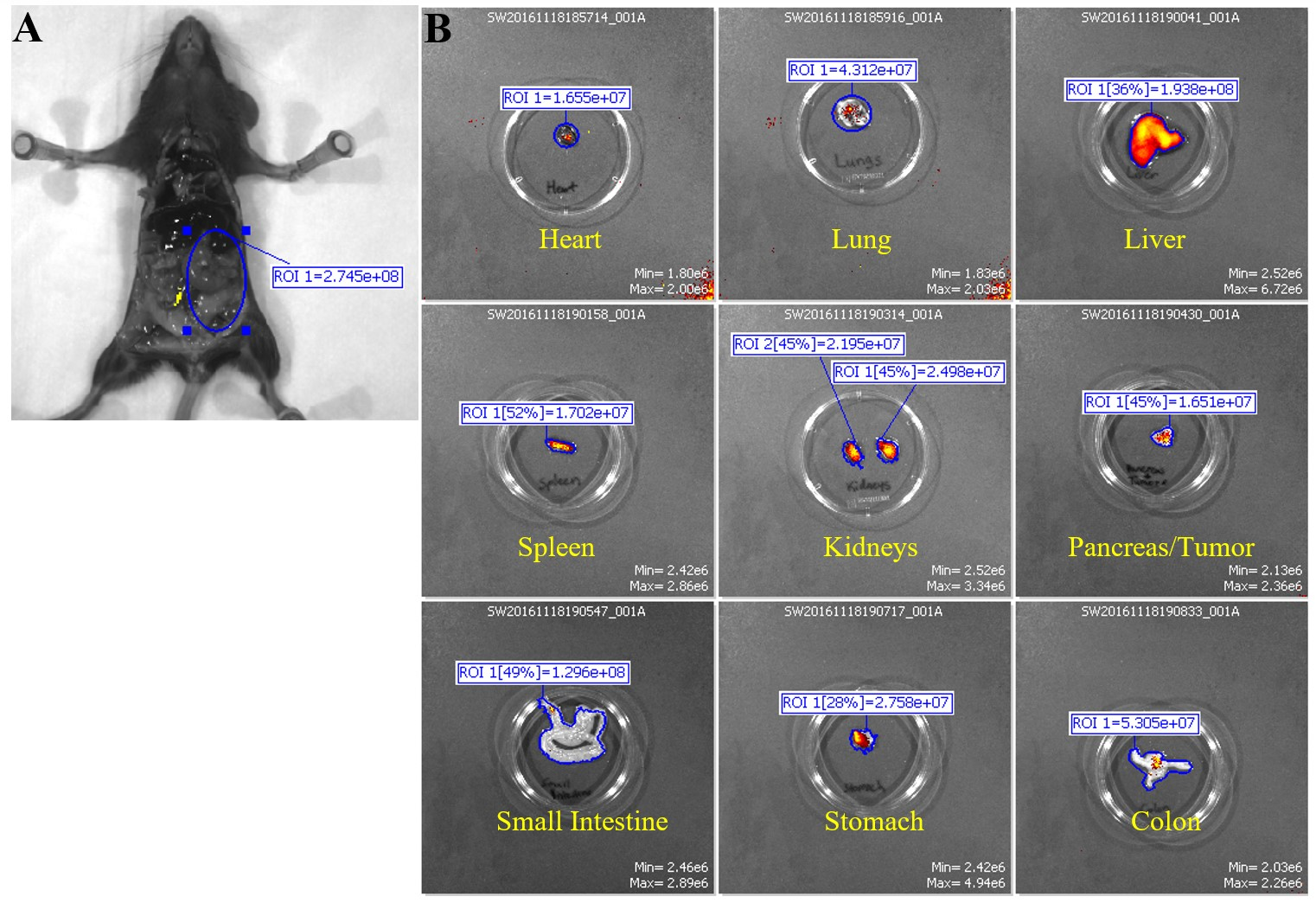

Supplement: S1 Fig — Representative images are shown. (A) The mouse is imaged with filter pair ICG on the IVIS Spectrum. Background has been removed and the ROI measurement for the area where tumor would have been present is shown. (B) Organs from mouse are imaged individually in the IVIS Spectrum. Intensity of the red-yellow fluorescence in ROI measurements indicate background levels for each organ. (TIF) [file pone.0193260.s001.tif]

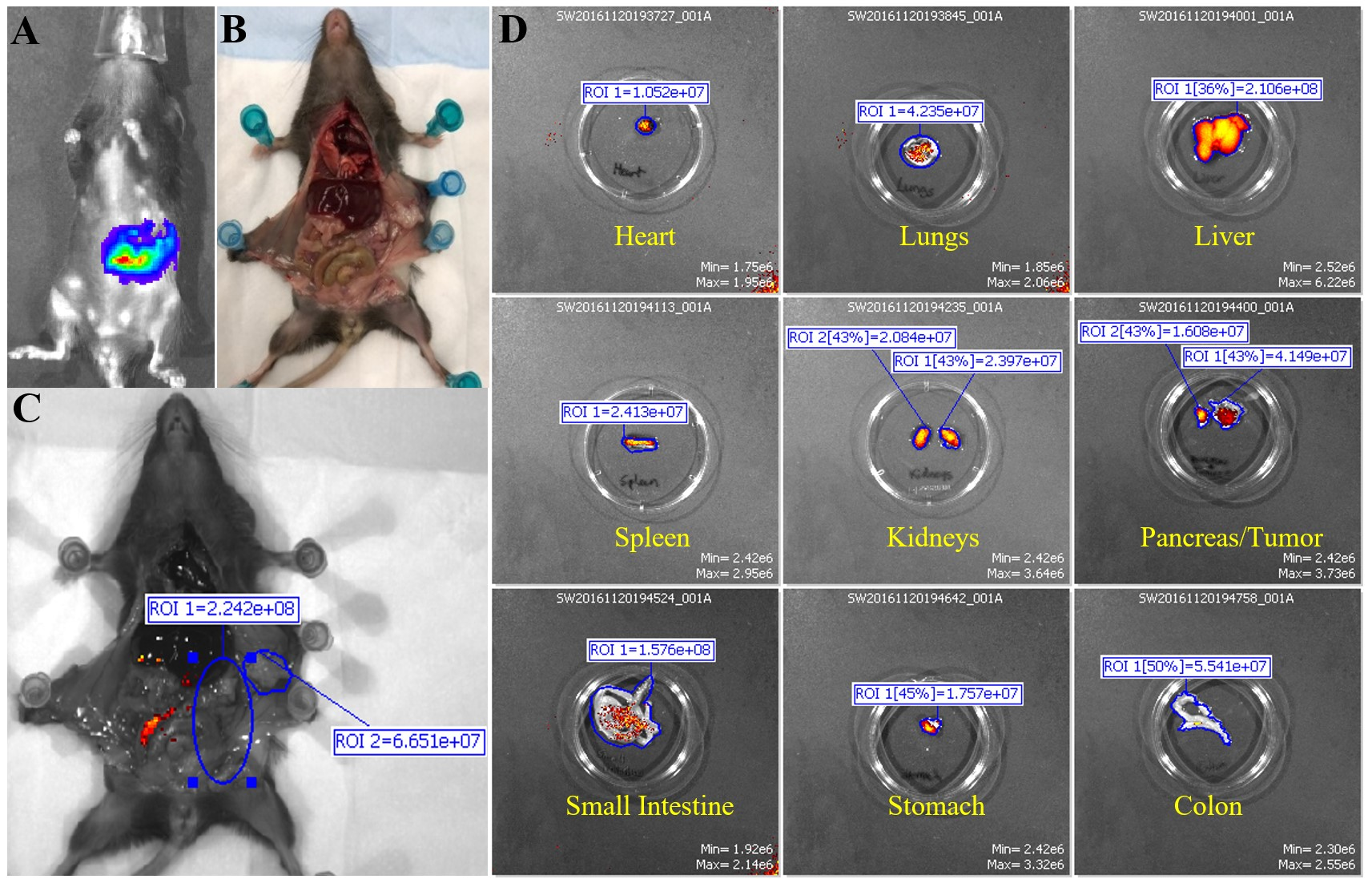

Supplement: S2 Fig — Representative images are shown. (A) Bioluminescent image of tumor. Rainbow indicates tumor site. (B) Photograph images of mouse to show location of tumor. (C) The mouse is imaged with filter pair ICG on the IVIS Spectrum. Background has been removed and the ROI measurements for the area where tumor is present and would have been present are shown. (D) Organs from mouse are imaged individually in the IVIS Spectrum. Intensity of the red-yellow fluorescence in ROI measurements indicates background levels for each organ. (TIF) [file pone.0193260.s002.tif]

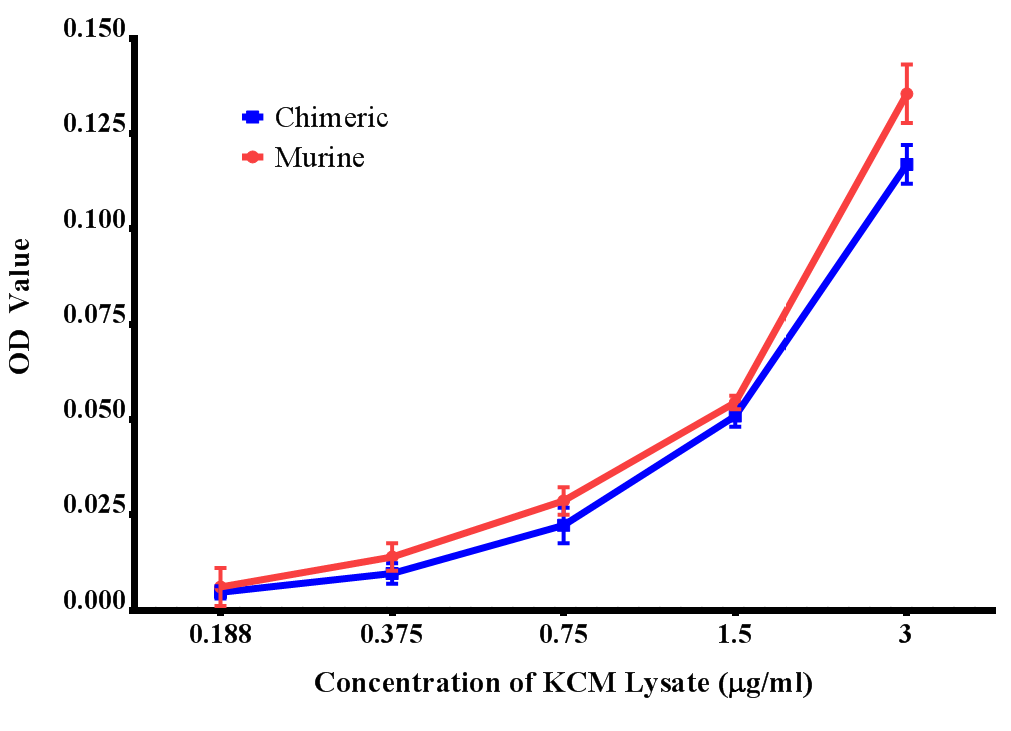

Supplement: S3 Fig — The binding profiles of mTAB004 (red) and cTAB004 (blue) were determined by ELISA and the OD values graphed against concentrations of KCM lysate. (TIF) [file pone.0193260.s003.tif]

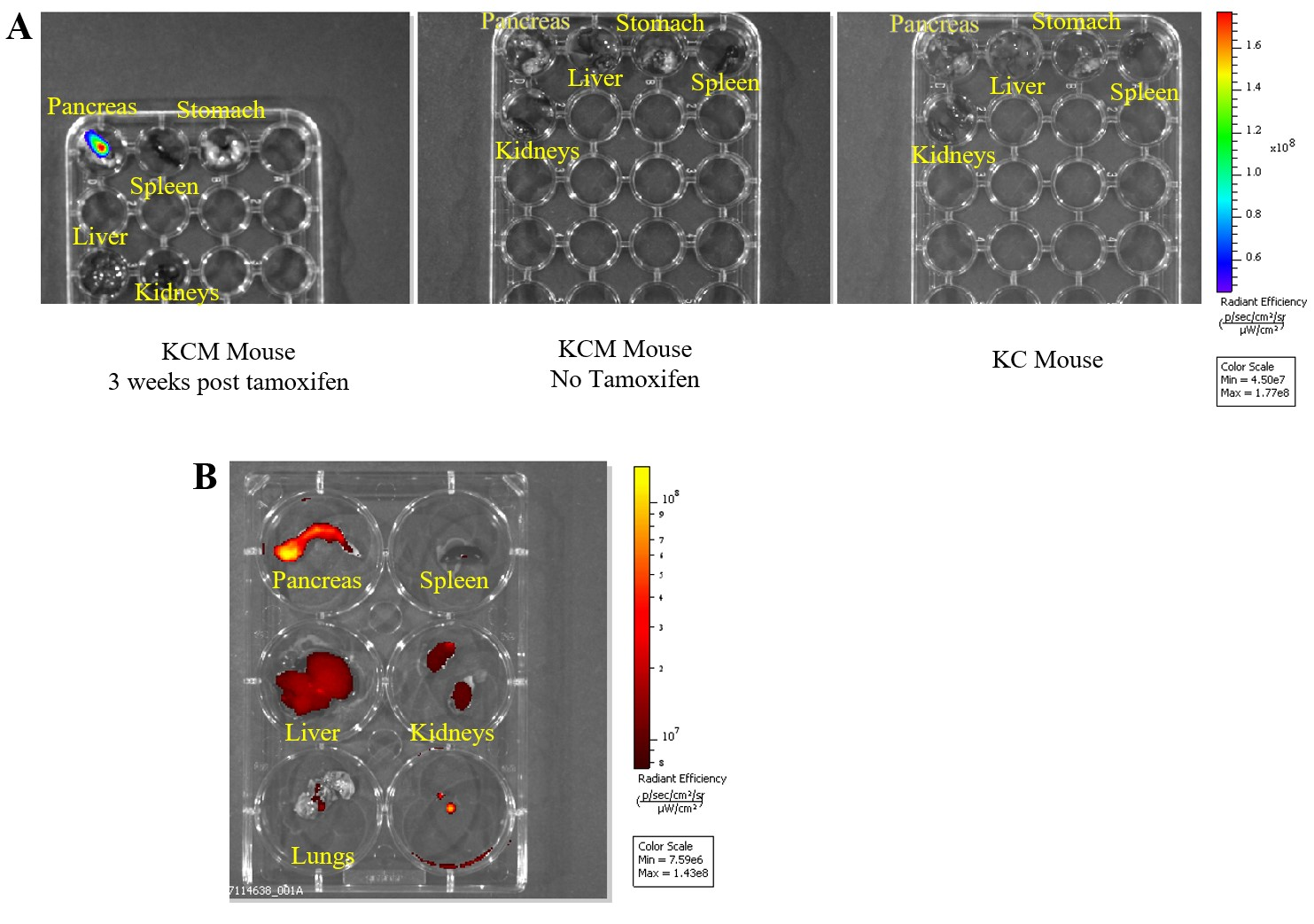

Supplement: S4 Fig — Representative images are shown. (A) IVIS images with ICG filter pair of organs from KCM Spontaneous mouse 3 weeks post tamoxifen induction, KCM Spontaneous mouse w/o tamoxifen, and a KC mouse. B) Organs from a KCM Spontaneous mouse 11 weeks post tamoxifen induction. Left–photograph of organs, Middle–Legend, Right–IVIS images with ICG filter pair. Intensity of the red-yellow fluorescence in ROI measurements indicates background and antibody accumulation for each organ. (TIF) [file pone.0193260.s004.tif]

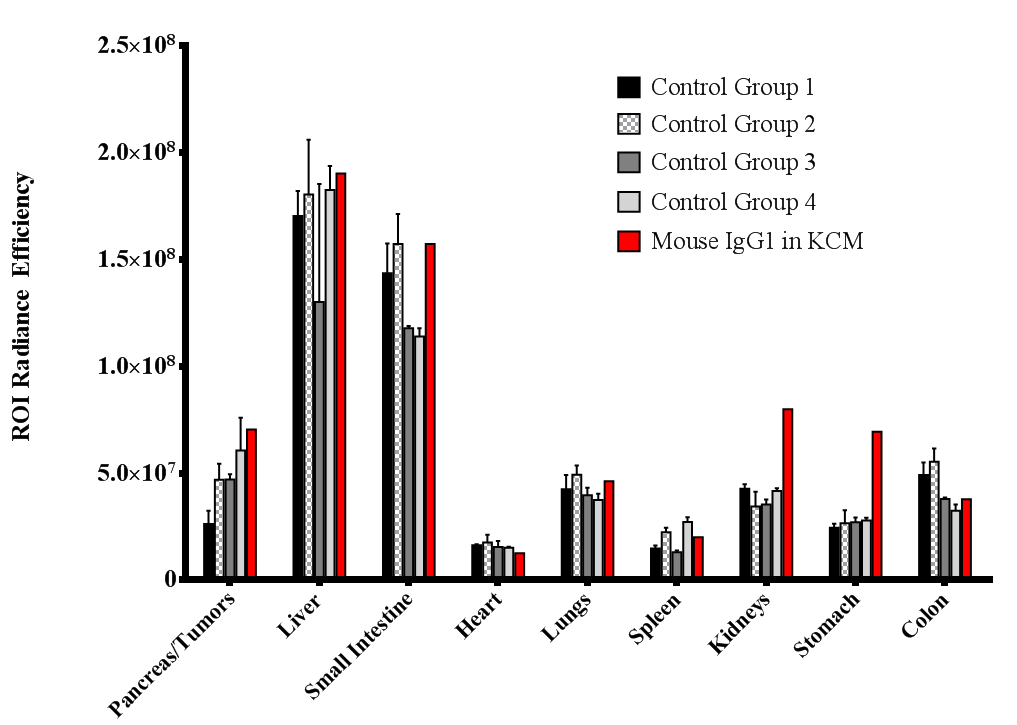

Supplement: S5 Fig — The ROI radiance efficiency values for organs from control groups were quantified used Living Image software. Data shown is mean ±SEM (n = 3), except for Mouse IgG1 in KCM group (n = 1, only 1 mouse was available for this experiment). (TIF) [file pone.0193260.s005.tif]
